# Do different adhesives influence the color stability and fluorescence of composite restorations after aging?

**DOI:** 10.1590/0103-6440202305504

**Published:** 2023-12-22

**Authors:** Claudia Simoes de Souza, Thamirys da Costa Silva, Mariana Sati Cantalejo Tsutsumi, Glivia Queiroz Lima, Mariana Elias Queiroz, Henrico Badaoui Strazzi-Sahyon, Ana Teresa Maluly-Proni, Andre Luiz Fraga Briso, Paulo Henrique dos Santos

**Affiliations:** 1 Department of Preventive and Restorative Dentistry, Araçatuba School of Dentistry - Sao Paulo State University, Araçatuba, SP, Brazil.; 2 Department of Dental Materials and Prosthodontics, Araçatuba School of Dentistry - Sao Paulo State University, Araçatuba, SP, Brazil.; 3 Department of Prosthodontics and Periodontology, Bauru School of Dentistry - University of Sao Paulo, Bauru, SP, Brazil.; 4 Dental Research Institute - Restorative Dentistry. Faculty of Dentistry - University of Toronto, Toronto, ON, Canada

**Keywords:** composite resins, dentin-bonding agents, color, fluorescence

## Abstract

This study aimed to evaluate the influence of dental adhesive color on the chromatic stability and fluorescence intensity of composite resin restorations of different thicknesses. Ninety bovine enamel samples were obtained and restored with resin composite varying thicknesses of restorative material and enamel (1.0 mm enamel and 1.0 mm composite; 1.5 mm enamel and 0.5 mm composite; 0.5 mm enamel and 1.5 mm composite). The restorations were made of composite resin (Opallis E-bleach H) using different types of dental adhesives: Ambar, Ambar APS, and Single Bond Universal (n=10). The samples were subjected to color measurement tests in a spectrophotometer using CIEDE2000 and fluorescence intensity measurements before and after aging in a red wine coloring solution. Data were subjected to analysis of variance (ANOVA) and Tukey's test (α = 0.05). There were no statistically significant changes in color stability or fluorescence intensity for restorations made of different materials or thicknesses (p>0.05). Single Bond Universal showed greater color stability at 0.5 mm thickness (ΔE00 = 4.4 ± 1.6) compared to other thicknesses of the same material (p=0.003), as well as a greater difference in fluorescence intensity after aging at 1.5 mm thickness (-414.9 ± 103.8) compared to other materials (p=0.0002). Overall, it was concluded that the different adhesive systems did not influence the color stability and fluorescence of restorations of different thicknesses.

## Resumo

O objetivo deste estudo foi avaliar a influência da cor do adesivo dental na estabilidade cromática e intensidade de fluorescência das restaurações de resina composta de diferentes espessuras. Foram obtidos noventa amostras de esmalte bovino que foram restaurados com resina composta, variando a espessura do material restaurador e do esmalte (1,0 mm de esmalte e 1,0 mm de compósito; 1,5 mm de esmalte e 0,5 mm de compósito; 0,5 mm de esmalte e 1,5 mm de compósito). As restaurações foram feitas de resina composta (Opallis E-bleach H) utilizando diferentes tipos de adesivos odontológicos: Ambar, Ambar APS, e Single Bond Universal (n=10). As amostras foram submetidas a testes de medição de cor em um espectrofotômetro usando CIEDE2000 e medições de intensidade de fluorescência antes e depois do envelhecimento em uma solução de coloração de vinho tinto. Os dados foram submetidos à análise de variância (ANOVA) e ao teste de Tukey (α = 0,05). Não houve mudanças estatisticamente significativas na estabilidade da cor ou intensidade de fluorescência para restaurações feitas de diferentes materiais ou espessuras (p>0,05). O Single Bond Universal mostrou maior estabilidade de cor a 0,5 mm de espessura (ΔE00 = 4,4 ± 1,6) em comparação com outras espessuras do mesmo material (p=0,03), bem como uma maior diferença na intensidade de fluorescência após o envelhecimento a 1,5 mm de espessura (-414,9 ± 103,8) em comparação com outros materiais (p=0,0002). Em geral, concluiu-se que os diferentes sistemas adesivos não influenciaram a estabilidade de cor e a intensidade de fluorescência das restaurações de diferentes espessuras.

## Introduction

Advances in dentistry have revolutionized important aspects of restorative and preventive care. With the development of adhesive materials, it is no longer necessary to prepare retentive walls to retain the material ^1^. The quality of the bond interface composed of the dental tissue and the adhesive system plays a fundamental role in the longevity of restorations ^2^. Considering this, obtaining a high degree of conversion of this material is essential, as this factor directly impacts the mechanical properties and degradation of this layer ^2^.

One of the factors that directly impact the degree of conversion of light-activated materials is the photo initiator system ^2^. Furthermore, the photo initiator is an important factor regarding the color and color stability of restorations ^3^. It is known that the most common initiating system for resin-based materials is camphoroquinone in conjunction with tertiary amine ^2^ ^,^ ^3^ ^,^ ^4^. One of the most significant advantages of camphoroquinone is the compatibility of its light absorption spectrum with the emission of most common lighting units (second-generation LEDs) ^5^. On the other hand, the yellowish appearance and color change in the material over time, resulting from the oxidation of the co-initiator, are among the limitations of camphorquinone ^2^ ^,^ ^3^. This yellowing affects not only the resin composite but also the adhesive itself. It could potentially impact the final aesthetic appearance of restorations, as previous studies suggest that the color of adhesives can influence the color of restorations ^3^ ^,^ ^6^ ^,^ ^7^.

To address this problem, the dental industry is reducing or replacing camphoroquinone with other photo initiators. An example of this is the APS system (Advanced Polymerization System APS), where camphoroquinone is found in smaller quantities and is only used to initiate the polymerization reaction ^8^. This decrease in the amount of camphoroquinone, facilitated by the APS system, would render the adhesive colorless, resulting in greater color stability, increased curing depth, and the ability to work under ambient light, which is limited by the concentration of conventional photo initiators ^9^.

The final staining of a restoration is not only influenced by the color of the restorative composite but also by the comprehensive optical properties of all the layers and materials that make up the restoration, particularly in thinner restorations or in cases of direct resin composite veneers ^10^. The color of the substrate, the degradation of the bonding layer ^7^, the opacity and the thickness of the restorative materials ^11^, as well as the appropriate light-curing process, are also factors that could influence the final color of restorations ^12^. In general, a 2.0-mm-thick resin composite layer would be sufficient to mask an unfavorably colored tooth substrate ^10^. However, in thinner restorations, doubts remain regarding the effect of different adhesive materials on the final esthetic quality of the restoration. Dental adhesives could, to a lesser degree, influence the color of resin composite restorations with a thickness of 0.7 mm ^13^.

Therefore, this study aimed to analyze the influence of adhesive color on resin composite restorations of different thicknesses and to evaluate the color stability and fluorescence intensity during the aging process. The null hypotheses tested were: 1) there would be no difference in color stability and fluorescence intensity of resin composite restorations by varying the type of adhesive used; 2) there would be no change in color stability and fluorescence intensity by varying the thickness of the resin composite used; and 3) there would be no difference in staining (a*, b*, and L* axes) between the different types of adhesives used.

## Materials and methods

The variables under consideration were resin composite thickness (1.0 mm, 1.5 mm, and 0.5 mm) and dental adhesives: Ambar conventional adhesive system without APS (FGM, Joinville, SC, BR); Ambar conventional adhesive system with APS - colorless (FGM, Joinville, SC, BR); Single Bond Universal adhesive system (3M ESPE, St. Paul, MN, USA). Ninety bovine enamel samples were prepared and restored with resin composite and distributed into nine groups according to the thickness and adhesive system used (n=10). The samples were subjected to color measurement tests in a spectrophotometer (Shimadzu, Kyoto, JP) using the CIEDE2000 system and fluorescence intensity measurements before and after aging in a red wine solution. Isolated samples of the adhesives (5.0 × 2.0 mm) were also analyzed for color parameters. The three basic principles of experimentation were followed (repetition, randomization, and blocking).

### Sample preparation

Forty-five bovine incisors were obtained from animals aged 24-36 months. After extraction, the teeth were mechanically cleaned with periodontal curettes and subjected to prophylaxis with pumice and water. To prevent bacterial proliferation, the cleaned teeth were preserved in a physiological saline solution containing 0.1% thymol and stored in a refrigerator at a temperature of approximately 4°C until the start of the experimental phase. To standardize the initial color of bovine teeth, the color of the teeth was previously assessed using a Vita Easy Shade spectrophotometer (VITA Zahnfabrik, Bad Säckingen, Germany). After cleaning, the roots of all teeth were separated from the crown at the cemento-enamel junction.

The crowns were attached to a device coupled to the platform of a bench drill FGC-16 model (Ferrari, São Paulo, SP, BR), and with a diamond tip for glass cutting 8.0 mm in diameter (Dinser Diamond Tools Ltda, Sacomã, SP, BR), with constant irrigation, two samples of 6.0 mm diameter of bovine teeth were obtained from each incisor, for a total of 90 specimens. The 90 obtained samples were randomly divided into 30 samples for each enamel thickness.

After this stage, all discs were manually polished using #320 and #600 grit silicon carbide papers (Buehler Ltd., Lake Bluff, IL, USA), and dentin was removed until the enamel had a thickness of 1.5 mm, 1.0 mm, or 0.5 mm, measured with a digital caliper (Mitutoyo Sul América Ltda, Jundiaí, SP, BR), with 30 discs for each thickness. The discs were then divided into three groups according to the type of adhesive system used, totaling nine experimental groups (n=10) according to the adhesive system and resin composite/enamel thickness.

Group AU: The surfaces of the enamel disks were etched with 37% phosphoric acid Condac 37 (FGM, Joinville, SC, BR) for 30 s, washed, and air sprayed. On the etched surface, Ambar etch-and-rinse adhesive (FGM) was applied actively in two layers for 10 s, air sprayed for 10 s to evaporate the solvent, and light-cured for 10 s using the multiwave light curing unit VALO® Cordless (Ultradent, South Jordan, UT, USA) using a standard mode with an irradiance of 1000 mW/cm^2^. The composite restorations were made with Opallis E-bleach H resin (FGM, Joinville, SC, BR) on the enamel surface, with varying thicknesses of the material. The final thickness of the specimens was always 2.0 mm made by a combination of 0.5, 1.0, and 1.5 mm of the enamel and resin composite of 1.5, 1.0, and 0.5 mm. A polyester strip and glass slide were positioned over the set-in to standardize the surface texture of the restoration and the resin composite was light-cured for 20 s. Final polishing was performed in a polisher with #1200 grit sandpaper and 1 µm diamond suspension applied to a felt disc (Arotec APL4, Cotia, SP, Brazil) for 60 s.

Group AU-APS: The same AU group protocol was used. However, the conventional Ambar APS adhesive (FGM) was used, as previously described.

Group SBU: The same adhesion process protocol was performed as for Group AU. Adhesive Single Bond Universal (3M) was used in the same way as described previously. The materials, composition, and lot numbers used in this study are listed in Box 1.

**Box 1:**
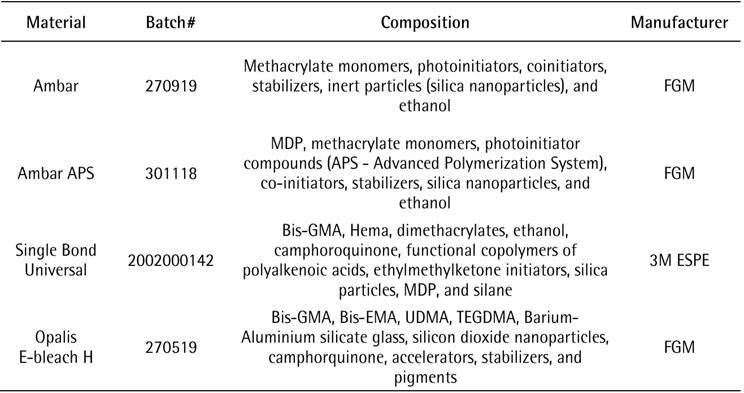
Identification of materials

### Color analysis

Specimens were chromatically analyzed immediately after polymerization using a visible ultraviolet reflection spectrophotometer*, Model UV-2450 (Shimadzu, Kyoto, JP), with color evaluation calculated using the CIEDE 2000 System. The CIEDE2000 formula employs the concepts of hue and Chroma to calculate the measured color difference that is as close as possible to the human eye's perception limits in the CIE Lab color space (L*a*b* color space). Values of tone or color parameters (a*: red-green ratio; b*: yellow-blue ratio; L*: black-white ratio) were recorded by the instrument. Specific adjustments could be applied to the differences in luminosity ΔL*, saturation ΔC*, and hue ΔH* using the *S*_*L*_ , *S*_*C*_ , and *S*_*h*_ coefficients, respectively. The adjustment factors *S*_*L*_ , *S*_*C*_ , and *S*_*h*_ , include the effects of L*, C* saturation, and H* hue angle. Consequently, the computation integrates the boundary perception properties of the human eye, namely, saturation dependence, hue dependence, and luminance dependence, in the CIE Lab color space (L*a*b* color system), supporting Munsell’s notions ^14^.

To perform the measurements, black silicon supports with a diameter of 6.0 mm and a thickness of 2.0 mm was made for the discs to perfectly fit, standardizing the position of the specimen and ensuring that the light beam always reached the same place. A demarcation was performed on the posterior part of each specimen to enable insertion into the color analysis device. For each sample, three-color analysis readings were obtained, and the arithmetic mean of the values was calculated.

L* a* b* color parameter readings were also taken on individual disks of different adhesive systems using the same parameters described above (n=5).

### Fluorescence intensity analysis

Fluorescence intensity measurements were performed on all the samples using a fluorescence spectrophotometer RF-5301 PC (Shimadzu Corp., Kyoto, JP). Specimens were fixed in silicon support and received the excitation beam at the center of the sample (400-600 nm), with emission and excitation "slits" of 2.5 nm opening. Data were registered in the computer attached to the spectrofluorometer in the form of a graph, and the mean fluorescence intensity values between 420 nm and 470 nm, corresponding to the visible light spectrum between violet and blue, were calculated. Three readings were recorded for each specimen, and an arithmetic mean was obtained.

### Aging of specimens

After initial color and fluorescence measurements, samples were individually immersed in flasks containing 5.0 ml of red wine (Quinta do Morgado, red table wine, pH 3.6, Serra Gaúcha, Brazil) for 28 days and kept in an incubator (ECB-2, Adamo Products for Laboratory Ltda, Piracicaba, São Paulo, Brazil) at 37°C ^15^ ^,^ ^16^. Flasks were sealed to prevent evaporation of the solution, which was changed weekly.

After the aging process, new color and fluorescence measurements were performed in the same way as described previously. The color stability (ΔE_00_) of the restorations was calculated using the following formula:

$$∆E_{00}=(K_{L}:K_{c}:K_{H})={\left[{\left(\frac{∆L´}{K_{L}S_{L}}\right)}^{2}+{\left(\frac{∆C^{'}}{K_{c}S_{c}}\right)}^{2}+{\left(\frac{∆H^{'}}{K_{H}S_{H}}\right)}^{2}+\;R_{T}\left(\frac{∆C^{'}}{K_{c}S_{c}}\right)\left(\frac{∆H^{'}}{K_{H}S_{H}}\right)\right]}^{1/2}$$

where *ΔL', ΔC'*, and *ΔH'* are the parameter differences of the brightness, chroma, and hue of the samples, respectively, using CIEDE2000. The functions *S*_*L*_ *, S*_*C*_ *,* and *S*_*h*_ adjust the color differences of the coordinates L', a', and b'. The parametric factors (KL, KC, and KH) are the correction terms for the experimental conditions. Finally, the rotation function (RT) describes the interaction between the chroma and hue differences in the blue region. The stability of fluorescence intensity was measured as the difference between the final fluorescence intensity and the initial fluorescence intensity of each sample.

### Statistical Analysis

Color change data of restorations, fluorescence intensity, and color analysis of the adhesives used were tested for normality (Shapiro-Wilk test) and homogeneity of variance (Bartlett test). The means of color change (ΔE_00_) and differences in fluorescence intensity were subjected to two-factor ANOVA and Tukey's test (α = 0.05). The color patterns of the adhesive systems were analyzed using a one-factor ANOVA and Tukey's test (α = 0.05).

## Results

Color change values of different thicknesses of resin composite restoration and different adhesive systems used are described in Table 1. There were no statistically significant differences in color change among the restorations built with the three different adhesive systems, regardless of the resin composite thickness (p=0.052; p=0.117; p=0.951). When comparing the thickness, only when Single Bond Universal was used, the 0.5 mm restorations showed less chromatic alteration compared to the 1.0 mm and 1.5 mm thicknesses (p=0.003). For the other materials, there were no significant differences between the different restoration thicknesses (p=0.063; p=0.589).

**Table 1:** Color change values (ΔE_00_ ± standard deviation) of different thicknesses of restorations built with different adhesive systems.

	Single Bond Universal	Ambar	Ambar APS
0.5 mm	4.4 ± 1.6 Ab	4.4 ± 0.8 Aa	5.6 ± 1.2 Aa
1.0 mm	6.3 ± 0.5 Aa	5.2 ± 2.1 Aa	6.9 ± 2.2 Aa
1.5 mm	6.7 ± 1.9 Aa	6.3 ± 2.0 Aa	6.5 ± 4.1 Aa

Means followed by distinct letters, upper case in the row, and lower case in the column, show statistically significant differences (p<0.05).

The changes in the fluorescence intensity values of the different restorations are shown in Table 2. Comparing the different adhesive systems used, at a thickness of 1.5 mm, restorations made with Single Bond Universal showed a greater decrease in fluorescence intensity compared to other adhesives used (p=0.0002). For other thicknesses, there were no statistically significant differences between the materials (p=0.156; p=0.266). Comparing the different thicknesses, only restorations made with Ambar APS showed a lower change in fluorescence intensity for the 1.5 mm thickness compared to the 0.5 mm thickness (p=0.04). For the other materials, no differences were observed between the thicknesses studied (p=0.05; p=0.311).

**Table 2:** Mean and standard deviation of the differences in fluorescence intensity (before and after aging) of different thicknesses of composite restoration with different adhesive systems.

	Single Bond Universal	Ambar	Ambar APS
0.5 mm	-483.4 ± 80.5 Aa	-375.8 ± 144.5 Aa	-423.5 ± 128.8 Aa
1.0 mm	-465.2 ± 116.9 Aa	-348.3 ± 217.7 Aa	-375.9 ± 139.6 Aab
1.5 mm	-414.9 ± 103.8 Aa	-202.7 ± 91.2 Ba	-277.1 ± 98.9 Bb

Mean values followed by different letters, upper case in the row, and lower case in the column, show statistically significant differences (p<0.05).

**Table 3:** Average and standard deviation of color analysis patterns of the adhesive systems studied.

	L*	a*	b*
Single Bond Universal	40.9 ± 2.5 A	-5.8 ± 0.3 A	13.2 ± 1.9 A
Ambar	40.4 ± 2.0 A	-3.7 ± 1.0 B	6.4 ± 2.9 B
Ambar APS	29.7 ± 0.5 B	-2.6 ± 0.3 C	2.7 ± 0.4 C

The mean intrinsic color values of the adhesive systems, followed by different capital letters, indicate a statistically significant difference (p<0.05).

The values of the color coordinate L*, a*, and b* for each adhesive used are listed in Table 3. The L* and b* values of the Ambar APS adhesive were significantly lower than those of the other materials studied (p<0.0001). The a^*^ values presented by this adhesive were significantly higher than those of the other materials were (p<0.0001).

## Discussion

This study evaluated the color stability of resin composite restorations with different thicknesses and types of adhesive systems. The main difference observed in the adhesive systems used in this study was related to the photo initiator/co-initiator of these materials. The results of the study showed no difference in the color stability (ΔE_00_) of resin composite restorations after aging using different adhesive systems, but some differences in the fluorescence intensity were observed, rejecting the first null hypothesis of the research. The developed photo initiator in the Ambar APS adhesive system called the APS (FGM), has shown promise according to a previous study conducted by Oliveira et al.^3^, where ΔE was evaluated through the CIELab* system of adhesive systems on ceramic laminates. Ambar APS showed the lowest ΔE value after storage in distilled water at different times (24 h, 7 days, 30 days, and 12 months) because, in addition to the photo initiator, this adhesive system is free of Bis-GMA, a molecule that could be associated with pigmentation through its by-products. The manufacturer claims that new photo initiators and initiators were added to this material, which acts synergistically to reduce the amount of camphoroquinone required for this material ^(^ ^17^. However, the exact composition of this system has not been determined. Higher values of ΔE were observed in the Single Bond Universal adhesive system, which could be attributed to the presence of higher amounts of camphoroquinone and tertiary amines, as well as its lower pH (1.4 to 2.6), which is associated with higher hydrophilicity and water sorption, impairing the color stability of the material ^3^.

In the present study, samples were stored in red wine for pigmentation of restorations, as occurs in the oral cavity, where liquids and foods that could pigment the dental tissues over time are ingested ^16^. CIEDE2000 was used for the color stability analysis, as it is more up-to-date in terms of the perception of the human eye, allowing the variations between the calculated values and the perceptibility to be attenuated. Thus, the formula used was as close as possible to the limits of human perception due to the modifications applied to differences in brightness ΔL*, saturation ΔC*, and color hue or tint ΔH* ^18^.

Cortopassi et al. ^19^ conducted a study in which they observed the ΔE_00_ values of surface sealants and adhesive systems applied to a resin composite at different times after storage in red wine. Between the materials used, the Ambar APS adhesive system showed higher ΔE_00_ values, which led the authors to report that the presence of this new photo activation system could have impaired the degree of conversion of the adhesive system, which is correlated to the color stability of resin composites. After 72 h of storage, ΔE_00_ values of the materials used stabilized. In our study, the adhesive systems were not in intimate contact with the pigment solution, as in the study cited above, but the presence of hydrophilic monomers may have resulted in the sorption of this solution at the margin of the adhesive interface, leading to the pigmentation of this material.

A value of 0.8 of ΔE_00_ is a color change perceptible to trained eyes, such as those of dental surgeons. With ΔE_00_ values between 0.8 and 1.8, the color change is regarded as acceptable, and there is no need to change the restoration. Above 1.8, the restoration needs to be changed ^20^. All ΔE_00_ values found in this study were greater than 1.8 (Table 2), which clinically indicates the need for restoration replacement ^20^. However, it is important to note that the conditions used in the experiment to age the samples were intended to accentuate the pigmentation processes that occur in the oral cavity.

In terms of thickness, it was observed that there was a directly proportional increase in the value of ΔE_00_ and the thickness of the restorative material. Thus, the second null hypothesis of the study was rejected because there was a statistical difference between the 0.5 mm thickness and the 1.0 mm and 1.5 mm thicknesses for Single Bond Universal (p=0.003). This might be attributed to better photo activation of the adhesive system as a result of the smaller thickness of the restorative material, resulting in a higher degree of conversion of the restorative assembly ^21^ ^,^ ^22^. The light-activation of the resin restoration may have acted as an extra light-activation of the adhesive system. Being exposed to a longer photo activation time and a greater amount of light through a thinner restoration, the adhesive system could have presented better properties ^22^. On the other hand, the color change can also be attributed to greater resin thicknesses. Ardu et al. ^23^ evaluated the L*, a*, and b* coordinate values of different resins with different thicknesses, where the ΔE_00_ results also tended to increase as the resin composite thickness increased.

Fluorescence is a physical phenomenon in which a material absorbs ultraviolet (UV) light and emits visible light in the blue spectrum ^24^. In dental tissues, fluorescence is mainly determined by dentin due to the photosensitive organic components present in this tissue ^24^. Catelan et al. ^24^ reported in their study that all pigmenting substances tested decreased fluorescence intensity values, with the greatest reduction in this value being caused by red wine. The acidic pH of this solution affects the integrity of the material surface, resulting in a greater accumulation of pigment and consequently, a decrease in the fluorescence intensity. In the present study, the fluorescence intensity of the samples decreased after aging. Restorations with Single Bond Universal showed a greater decrease in fluorescence intensity compared to other materials at 1.5 mm thickness (p=0.0002), and among the thicknesses, only Ambar APS showed a lessened decrease in fluorescence intensity between 1.5 mm and 0.5 mm thickness (p=0.04). A trend was observed, where the thickness of the restorative material increased and the fluorescence intensity decreased (Table 2). This finding was reported by Tabatabaei et al. ^25^, who refer to this phenomenon as blackout, which occurs when there is an increase in the thickness of fluorescent materials. Absorption of fluorescent light occurs mainly by fluorescent particles present on the surface of the material, but there are other particles with the same properties in depth. Therefore, when surface particles reach their absorption threshold, light is absorbed by deeper particles, decreasing the fluorescence intensity of the material ^25^.

Because the values of L*, a*, and b* color coordinates of adhesive systems show a statistical difference when comparing materials, the third null hypothesis of the study was rejected. It was possible to observe that Single Bond Universal has the highest value of the b* coordinate (yellow and blue) (Table 2), which is probably related to the higher amount of camphoroquinone in this material, which has a chromatic group in its composition that is responsible for making this compound photoactive but also gives it a yellowish color ^3^. Ambar APS showed a lower b* value (yellow and blue), which could indicate the lower presence of camphoroquinone in its composition. However, manufacturers do not provide the exact composition of these materials. In the analyses performed, different colors of the adhesive systems did not influence the color stability and fluorescence intensity of the restorations.

Some limitations of this study include the inability to mimic processes occurring in the oral environment, such as the presence of saliva and brushing performed by the patient, which might result in increased stability of the optical characteristics of the materials. Therefore, as a result, in vitro and clinical studies should be extended to evaluate the properties of the materials due to constant innovations of materials in the dental market.

Despite the variation in the color of the adhesive systems employed, these materials did not yield a noticeable difference in the color change of the composite resin restorations when compared to each other. In cases of thicker restorations, the Single Bond Universal adhesive induced a more significant variation in restoration fluorescence compared to the other adhesives tested. In terms of thickness variation, enhanced color stability was observed in thinner restorations when utilizing the Single Bond Universal adhesive, as opposed to other thicknesses examined.
